# New Garden Rose (*Rosa* × *hybrida*) Genotypes with Intensely Colored Flowers as Rich Sources of Bioactive Compounds

**DOI:** 10.3390/plants13030424

**Published:** 2024-01-31

**Authors:** Nataša Simin, Nemanja Živanović, Biljana Božanić Tanjga, Marija Lesjak, Tijana Narandžić, Mirjana Ljubojević

**Affiliations:** 1 Faculty of Sciences, University of Novi Sad, Trg Dositeja Obradovića 3, 21000 Novi Sad, Serbia; 2 Breeding Company ‘Pheno Geno Roses’, Maršala Tita 75, 23326 Ostojićevo, Serbia; 3 Faculty of Agriculture, University of Novi Sad, Trg Dositeja Obradovića 8, 21000 Novi Sad, Serbia

**Keywords:** garden roses, *Rosa* × *hybrida*, polyphenols, antioxidant, FRAP, DPPH, acetylcholinesterase inhibition

## Abstract

Garden roses, known as *Rosa* × *hybrida*, hold a prominent position as one of the most important and economically valuable plants in horticulture. Additionally, their products—essential oil, rose water, concrete, and concentrate—find extensive use in the cosmetic, pharmaceutical, and food industries, due to their specific fragrances and potential health benefits. Rose flowers are rich in biologically active compounds, such as phenolics, flavonoids, anthocyanins, and carotenoids. This study aims to investigate the potential of five new garden rose genotypes with intensely colored flowers to serve as sources of biologically active compounds. Phenolic profile was evaluated by determination of total phenolic (TPC), flavonoid (TFC), and monomeric anthocyanins (TAC) contents and LC-MS/MS analysis of selected compounds. Antioxidant activity was evaluated via DPPH and FRAP assays, neuroprotective potential via acethylcholinesterase inhibition assay, and antidiabetic activity viaα-amylase and α-glucosidase inhibition assays. The flowers of investigated genotypes were rich in phenolics (TPC varied from 148 to 260 mg galic acid eq/g de, TFC from 19.9 to 59.7 mg quercetin eq/g de, and TAC from 2.21 to 13.1 mg cyanidin 3-*O*-glucoside eq/g de). Four out of five genotypes had higher TPC than extract of *R. damascene*, the most famous rose cultivar. The dominant flavonoids in all investigated genotypes were glycosides of quercetin and kaempferol. The extracts showed high antioxidant activity comparable to synthetic antioxidant BHT, very high α-glucosidase inhibitory potential, moderate neuroprotective activity, and low potential to inhibit α-amylase.

## 1. Introduction

In the realm of horticultural research, special attention is dedicated to the creation of new genotypes of flowering plants with intense flower colors, including red, violet, and purple, as their beauty adds a burst of life and vibrancy to any garden. Garden roses (*Rosa* × *hybrida*) stand out as specimens with captivating beauty and a rich palette of intense colors.

The pigments responsible for the red, violet, and purple hues of rose petals are primarily anthocyanins, a subgroup of flavonoid glycosides characterized by the presence of flavylium cation. Anthocyanins are water-soluble pigments known for their role as natural pigments in plants, contributing to the red, purple, blue, and violet colors of the flowers, depending on the pH environment. Beyond contributing to coloration, plant phenolics, particularly anthocyanins, contribute to the potential health benefits provided by plants. Numerous studies have suggested that anthocyanins derived from rose petals showcase robust anti-inflammatory, antioxidant, anticancer, antimicrobial, and antiallergic properties [1]. In addition to anthocyanins, rose hips and petals are rich in other polyphenols, essential oil, essential fatty acids, minerals (Ca, Mg, K, S, Si, Se, Mn and Fe), and vitamins A, C, and E [2]. Our recent findings supported the hypothesis that rose petals of new cultivars of *Rosa* × *hybrida* are a rich source of biochemically active compounds and have high antioxidant and neuroprotective activity [3]. Thus, besides their role in ornamental horticulture, roses could potentially find applications in various industries, including the cosmetic, pharmaceutical and food industry.

**Figure 1:**
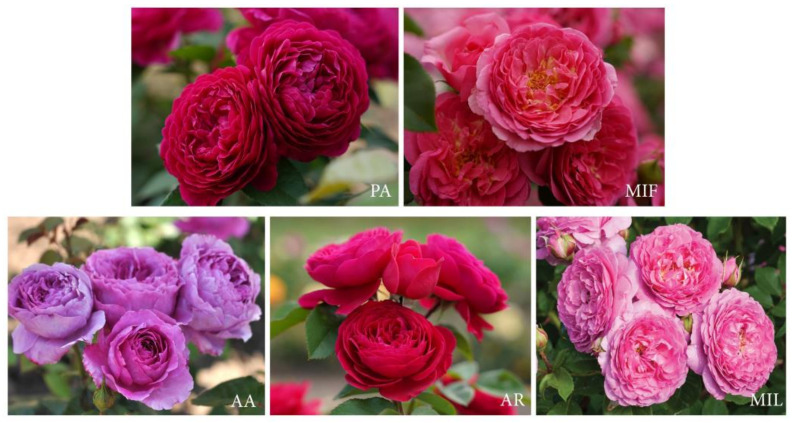
Investigated rose genotypes. PA—‘Pure Aroma’; MIF—‘Mina Frayla’; AA—‘Adore Aroma’; AR—‘Andre Rieu’; MIL—‘Mileva Frayla’.

Over the past decade, the ‘Pheno Geno Roses’ company in the Netherlands has successfully developed an extensive assortment of new garden rose varieties [4]. This achievement involved strategic hybridization, employing controlled pollination of parent plants with desirable traits and generating offspring with enhanced characteristics. A primary breeding objective was the identification of genotypes possessing favorable sensory attributes, particularly focusing on fragrance, with potential applications in the cosmetic and perfumery sectors. Standardized sensory analysis of the fragrance profiles was examined, extinguishing five out of the numerous genotypes evaluated (Figure 1) due to their notable fragrance and the presence of large, intensely colored flowers.

The objective of this research was to further enhance the value of these rose genotypes by analyzing the composition of biologically active polyphenols and exploring potential health benefits. Hence, the specific aims included the evaluation of the following: (a) morphological attributes concerning the abundance of flowering shoots and flowers, as well as the size and quantity of flower petals; (b) intensity of fragrance and the scoring using a sensory panel for major top, heart, and base fragrance notes, following the olfactory pyramid; (c) phytochemical composition related to polyphenols; (d) antioxidant capacity using The Ferric Reducing Antioxidant Potential (FRAP) and 2,2-diphenyl-1-picrylhydrazyl (DPPH)assays; (e) neuroprotective activity thought determination of the potential to inhibit acetylcholinesterase (AChE-IP); and (f) antidiabetic effect thought α-amylase and α-glucosidase inhibitory potential determination. Phytochemical profile determination included analysis of total phenolics (TPC), flavonoids (TFC) and monomeric anthocyanins (TAC) contents, and quantification of individual phenolic compounds. 

Determining the chemical composition of new rose genotypes and assessing their biological activities will enable the identification of cultivars with the greatest potential for health benefits, alongside their valuable sensory properties. This will potentially enhance the market value of these genotypes, making them more attractive to potential customers from the cosmetic and pharmaceutical industries.

## 2. Results

### 2.1. Morphological Traits

Tetraploid (4n) garden rose cultivars included ‘Pure Aroma’ (PA), ‘Adore Aroma’ (AA), ‘Andre Rieu’ (AR), ‘Mina Frayla’ (MIF), and ‘Mileva Frayla’ (MIL).

The growth type labeled as shrub did not differ in the investigated cultivars, while the semi-upright growth habit was noted in ‘PA’ and ‘MIL’, and upright in the remaining rose cultivars (Table 1). Plant height reached from 69.6cm in ‘AA’ to 90.4 cm in ‘MIL’, showing a significant difference according to the statistical test. Leaf color varied from a medium in ‘AR’ and ‘MIL’ to dark intensity of green color in ‘PA’, ‘AA’, and ‘MIF’. Leaf anthocyanin coloration was present in all investigated genotypes while glossiness of the upper side was weak or very weak in three out of five genotypes. Leaf length and width also showed significant variation according to Tukey’s HSD test, taking values from 3.58 cm to 5.68 cm and 2.66 cm to 3.88 cm, respectively, with both properties being minimal in ‘MIF’ and maximal in ‘MIL’.

As to the flowering shoot qualitative characteristics (Table 2), shoots were absent in all rose cultivars except in ‘AA’ with 1.82 shoots on average. Correspondingly, flowering laterals were present in all other cultivars, achieving values from 1.62 and 1.64 in ‘MIF’ and ‘AR’ to 3.44 in ‘MIL’, and the number of flowers per lateral achieving values from 2.94 in ‘PA’ to 8.72in ‘MIL’. Flower type was uniform in all investigated cultivars, varying from medium purple and medium violet to medium purple red and dark purple. The color of the flower center was red–purple in all investigated cultivars except for ‘MIL’. The flower shape was rounded in ‘AA’, ‘MIL’, and ‘AR’, while irregularly rounded in others. Upper part profiles were described as flat in all five cultivars, while the lower part profile differed only for ‘MIL’ (flat), while concave in the rest of the cultivars. Fragrance was marked as strong (label 5) for all cultivars. Regarding the quantitative characteristics, the number of petals per flower significantly differed, taking values from 65.8 in ‘MIF’ to almost double in ‘AA’ (114.4 petals), with the petals achieving the highest length in ‘AR’ and the highest value for width in ‘PA’.

### 2.2. Sensory Evaluation

**Figure 2:**
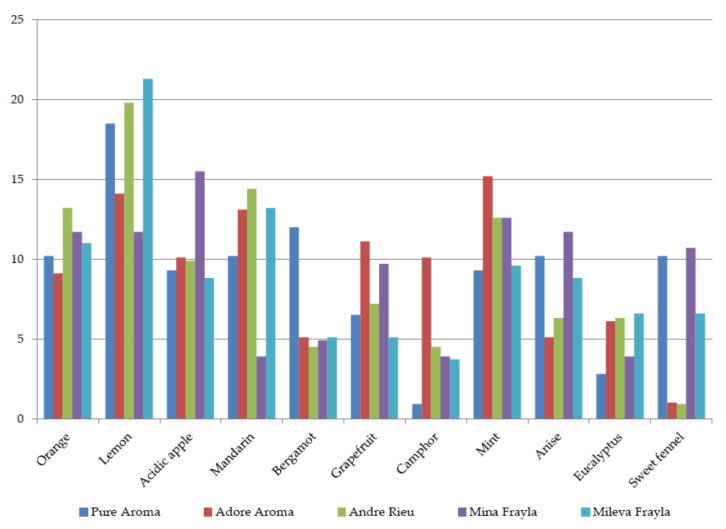
Panel scoring for top notes belonging to citrus or minty (aromatic) fragrance components.

**Figure 3:**
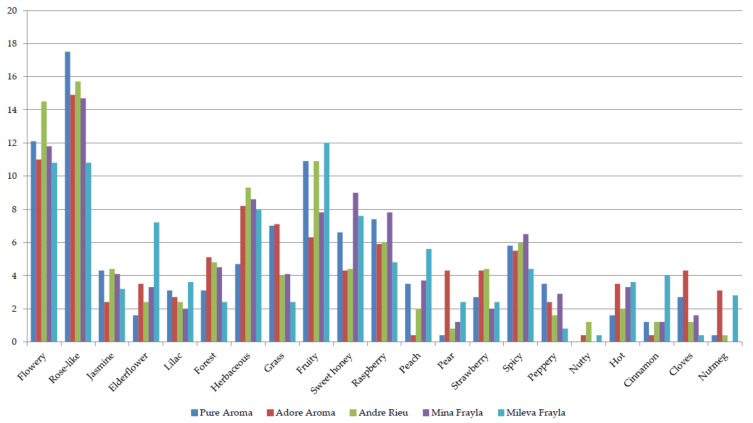
Panel scoring for middle notes belonging to floral, green, fruity, or spicy fragrance components.

**Figure 4:**
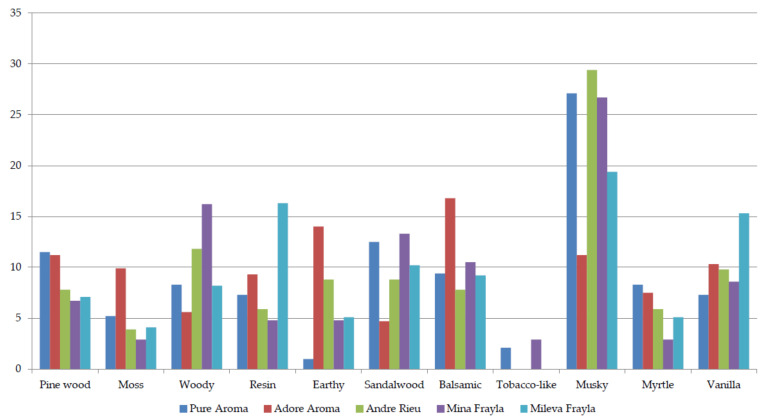
Panel scoring for base notes belonging to woody, earthy, or balsamic fragrance components.

**Figure 5:**
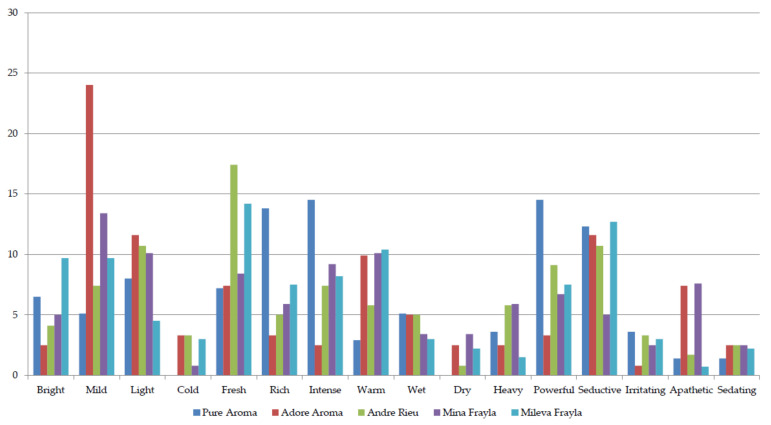
Panel scoring for general impression by the present fragrance components.

The fragrance was described as 5—strong in all five cultivars and further scored by the panelists for the presence of fragrance components (Figure 2, Figure 3, Figure 4 and Figure 5). Apart from receiving high scores for their overall fragrances, cultivars were characterized as a blend of various top, heart, and base notes. Specifically, the top notes showcased a prevalence of fruity aromas, with panelists linking them to various common fruit species such as orange, lemon, apple, mandarin, and grapefruit, supplemented with mint fragrance in ‘AA’ and anise and sweet fennel in ‘MIF’. Bergamot and eucalyptus scored lowest (mainly around 5%) while scores for sweet fennel varied from 0.9 to 10.7%. All cultivars seem to be very complex since panelists recorded notes from all 11 groups (Figure 2).

Middle notes belonging to 21 different categories showed a higher variation with only three groups reaching over the threshold of 10%—flowery(with elderflower dominant in ‘MIL’), fruity (among which raspberry and strawberry dominate) and typical rose-like notes. Spicy fragrances scored lowest, with the majority of components reaching less than 5%, while nutty and nutmeg (in a range 0–3%) can be considered as a mixed fragrance and not as a certain association with any of the investigated cultivars (Figure 3).

Regarding the base notes belonging to 11 different categories, eight exceeded the 10% threshold, with musky fragrance notably scored in all five cultivars (from 11.2% in ’AA’ to 29.4% in ‘AR’). Interestingly, different peaks can be noted according to Figure 4—woody fragrance in ‘MIF’, resin and vanilla in ‘MIL’, balsamic in ‘AA’, and musky in ‘PA’, ‘AR’, and ‘MIF’.

Panelists were asked to express the first emotional association upon the flower smelling, which yielded the results presented in the Figure 5. Most of the times panelists expressed association on mild in ‘AA’, fresh in ‘AR’, rich, intense, seductive, and powerful in ‘PA’ as well as fresh and seductive in ‘MIL’. Regarding ‘MIF’, associations were divided among mild, light, fresh, intense, and warm, with around 10% of the scores. Associations on cold, dry, irritating, or sedating effects were the least noted (below 5%).

### 2.3. Chemical Profile of Methanol Extracts of Rose Petals

The chemical analysis of methanol extracts from rose petals involved the determination of the phenolic profile through the measurement of total phenolics content (TPC), total flavonoids content (TFC), and total monomeric anthocyanins content (TAC) using spectrophotometric techniques. Furthermore, the quantification of quinic acid and 44 selected phenolic compounds was conducted using LC-MS/MS.

**Figure 6:**
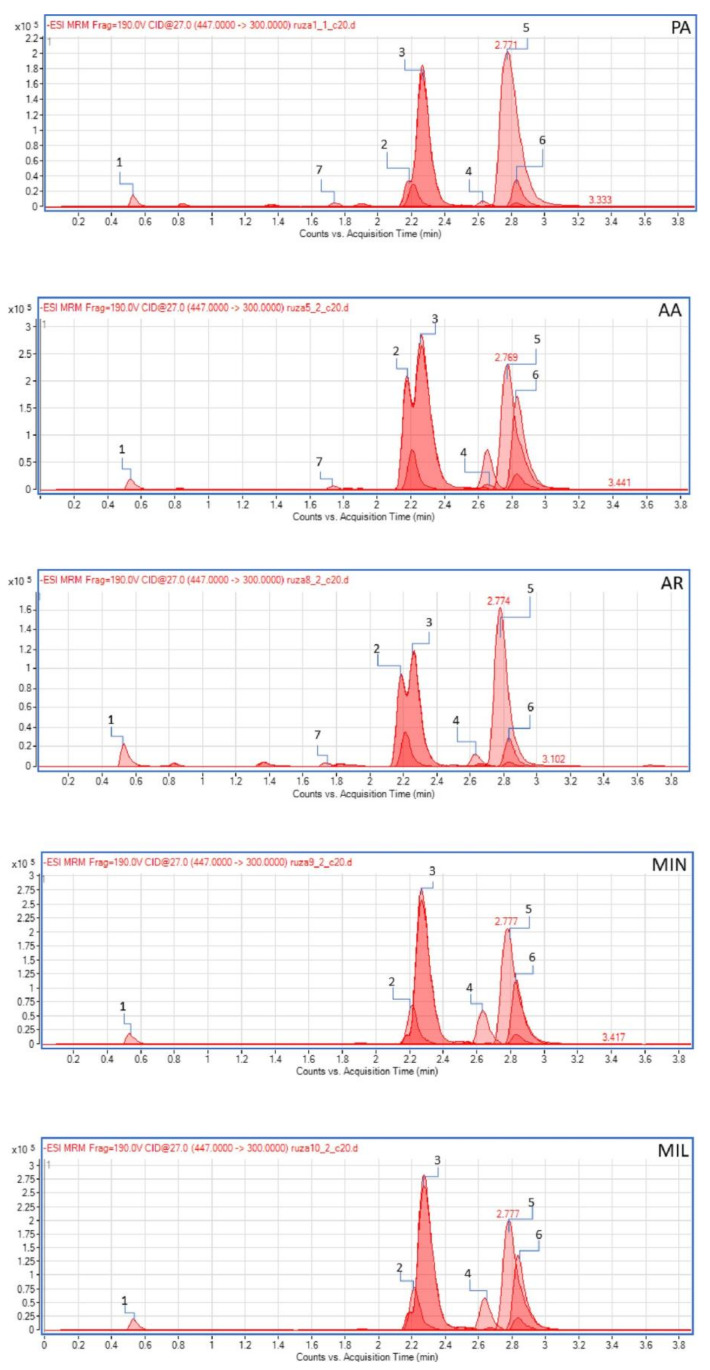
LC-MS/MS chromatograms of selected phenolic compounds in methanol extracts of rose petals.PA—Pure Aroma, AA—Adore Aroma, AR—Andre Rieu, MIN—Mina Frayla, MIL—Mileva Frayla. Only the most pronounces peaks are marked: 1—Quinic acid, 2—Quercetin-3-*O*-galactoside, 3—Quercetin-3-*O*-Glucoside, 4—Rutin, 5—Quercitrin, 6—Kaempferol-3-*O*-Glucoside, 7—p-Coumaric acid.

The results obtained indicated that the studied rose genotypes are rich in phenolic compounds (as depicted in Table 3 and Figure 6). Nevertheless, there was significant variability observed in TPC, TFC, and TAC among different genotypes. The samples AR and PA were the richest in TPC (260 mg GAE/g de and 249 mg GAE/g de, respectively), while the sample AA has the lowest TPC (148 mg GAE/g de). When expressed in terms of 1 g of fresh petal weight, the TPC ranged from 14.0 mg GAE/g fw in the AA sample to 26.8 mg GAE/g fw in the PA. The TFC of examined extracts ranged from 23.7 mg QE/g de to 59.8 mg QE/g de and decreased in the following order: AA > MIF > MIL > PA > AR. If expressed per gram of fresh petals, the TFC was in the range from 2.36 mg QE/g fw to 5.70 mg QE/g fw. The results obtained for TAC indicated that all samples contain a significant number of anthocyanins, ranging from 2.21 mg CE/g de in the MIL sample to 13.1 mg CE/g de in the AR genotype. If expressed per gram of fresh petals, the TAC ranged from 0.188 mg CE/g fw to 1.31 mg CE/g fw.

The results of LC-MS/MS quantitative analysis indicated that out of the 45 compounds targeted for quantification, only 20 were detected (Table 4). Corresponding chromatograms are shown on Figure 6. Quercetin 3-*O*-glycosides, specifically quercitrin (quercetin 3-*O*-rhamnoside), quercetin 3-*O*-glucoside, quercetin 3-*O*-galactoside and rutin (quercetin 3-*O*-rutinoside), as well as kaempferol-3-*O*-glucoside, were identified as the predominant flavonoids in all investigated samples. It was not possible to quantify individual amounts of quercetin 3-*O*-glucoside and quercetin 3-*O*-galactosid, but only the total amount (quercetin 3-*O*-glucoside + quercetin 3-*O*-galactosid), since their peaks in the chromatogram were overlapped. Flavonoid aglycones—catechin, quercetin and kaempferol—were also present in all samples, but in significantly lower amounts. The quinic acid content was found to be very high in all investigated samples, ranging from 13.4 mg/g de in AA genotype to 23.9 mg/g de in AR. Among benzoic acid derivatives, low amounts of protocatechuic (12.8–60.7 μg/g de) and gallic acids (21.7–36.4 μg/g de) were found. Chlorogenic and *p*-coumaric acids were the only hydroxycinnamic acids found in analyzed samples, but their quantities were very low (up to 5.98 μg/g de). The following compounds were analyzed but not detected in any of the examined extracts or were below the limit of quantitation in all samples: 2,5-dihydroxybenzoic acid, epigallocatechin gallate, aesculetin, caffeic acid, vanillic acid, syringic acid, umbelliferone, scopoletin, ferulic acid, sinapic acid, hyperoside, apiin, o-coumaric acid, myricetin, secoisolariciresinol, 3,4-dimethoxycinnamic acid, baicalin, daidzein, matairesinol, cinnamic acid, luteolin, genistein, apigenin, baicalein, and amentoflavone.

**Figure 7:**
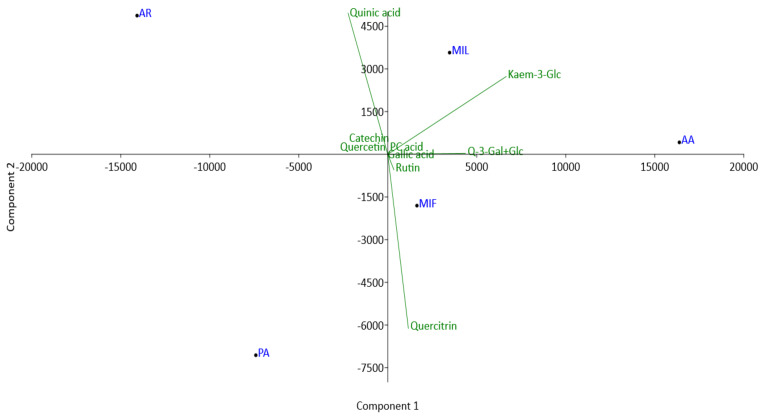
Biplot of a principal component analysis (PC1 vs PC2) analysis of 10 dominant compounds in rose petal methanol extracts. AA—‘Adore Aroma’; AR—‘Andre Rieu’; MIF—‘Mina Frayla’; MIL—‘Mileva Frayla’; PA—‘Pure Aroma’.

**Figure 8:**
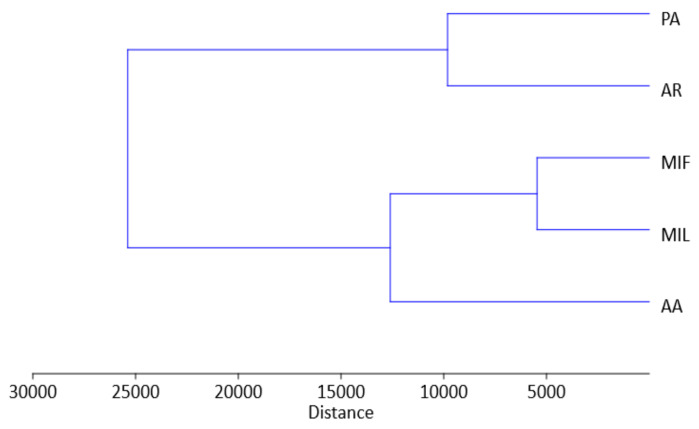
Dendrogram obtained using hierarchical clustering analysis of 10 dominant compounds in rose petal methanol extracts. AA—‘Adore Aroma’; AR—‘Andre Rieu’; MIF—‘Mina Frayla’; MIL—‘Mileva Frayla’; PA—‘Pure Aroma’.

Principal Component Analysis (PCA) was conducted on a dataset comprising 10 dominant compounds present in the extracts, each in amounts exceeding 10 μg/g (Figure 7). The first and second principal components (PC1 and PC2) accounted for 83.2% and 13.9% of the total variance, respectively, which indicates significant metabolic differences among the investigated rose genotypes. The analysis revealed a certain level of grouping of the samples AA and MIL in the upper right quadrants of the biplot. This could mainly be attributed to the high loadings of kaempferol-3-*O*-glucoside and quercetin-3-*O*-glucoside + quercetin-3-*O*-galactoside. A common feature for samples AR and PA is significantly lower amounts of these glycosides compared to the other samples. AR is separated in the left upper quadrant of the biplot due to extremely high content of quinic acid compared to other samples. The sample PA stands out from the others, primarily due to the significantly higher amount of quercitrin present in this sample. The sample MIF is characterized by moderate levels of all analyzed compounds and this is the main reason for separation of this sample in the lower right quadrant of the biplot. The similar conclusions can be made from the the dendogram obtainedusing hierarchical clustering analysis of the same data (using the Ward’s method, where closeness was measured by Euclidean distance, Figure 8). 

### 2.4. Methanol Extracts’ Antioxidant Activityof Rose Petals

The antioxidant capacity of methanol extracts from rose petals was evaluated using two assays: FRAP and DPPH assay.

All examined rose petal extracts in the current investigation demonstrated noteworthy antioxidant activity (refer to Table 5). A robust correlation was observed between the results of the two antioxidant assays carried out (R^2^ = 0.983). In the FRAP assay, the activity of the extracts was in the range from 132 mg AAE/g de to 209 mg AAE/g de. The strongest ferric ion reducing ability was expressed by PA and AR extracts. The IC_50_ values in the DPPH assay fell within a very narrow range (17.7–27.8 μg/mL) for all investigated genotypes, with the highest activity observed in the extracts of PA and MIL.

### 2.5. Methanol Extracts’ Neuroprotective Activity of Rose Petals

The neuroprotective activity of the rose petal extracts was estimated by measuring their ability to inhibit acetylcholinesterase (AChE). Since alkaloid physostigmine (eserine), a highly potent AChE inhibitor, was used as a positive control, the results were expressed as nanograms of eserine equivalents per gram of dry extract (EE/g de; Table 5). The AChE inhibitory activity of the extracts at a concentration of 50 µg/mL was considerable and ranged from 29% to 40%. When expressed in eserine equivalents, anti-AChE activity ranged from 10.2–18.3 ng EE/g de. The highest activity was exhibited by the extract of PA genotype (18.3 ng EE/g de).

### 2.6. Methanol Extracts’ Antidiabetic Activity of Rose Petals

The methanol extracts of rose petals were assessed for their potential antidiabetic properties through the evaluation of their inhibitory effects on α-amylase and α-glucosidase enzymes. In the α-amylase assay, the results were expressed as the percentage of inhibition for the extracts at a concentration of 278 µg/mL and as mg of acarbose equivalents (ACAE)/g de (Table 5). The results in α-glucosidase assay were expressed as percentage of inhibition for the extracts at a concentration of 2.5 µg/mL and as g of ACAE/g de (Table 5). The extracts expressed much higher inhibitory activity towards α-glucosidase than against α-amylase. Even at a very low concentration (2.5 µg/mL), samples PA and AR achieved nearly 90% inhibition of α-glucosidase. Samples AA, MIL and MIF exhibited slightly lower inhibitory activity against α-glucosidase (47.2–57.1%). The extracts exhibited significantly higher activity than the well-known α-glucosidase inhibitor, acarbose, as evident from the results expressed as g ACAE/g de. Inhibitory potential of the extracts against α-amylase was negligible (0–13.6% of inhibition at a concentration of 167 µg/mL).

## 3. Discussion

The creation of new rose genotypes can be driven by various factors. One of the most important drivers is to fulfill market demands by developing roses with enhanced characteristics, such as color variations, fragrance, size, and petal structure, to meet specific aesthetic preferences [5]. Other driving forces include the creation of new genotypes with extended blooming periods and resistance to common diseases, and which are well-adapted to specific climatic conditions or regions [5]. Beyond ornamental purposes, roses are essential raw materials in the cosmetic and pharmaceutical sectors, which underscore the economic and cultural significance of these plants [6]. Among all plant parts, rose flowers and fruits (rose hips) are the most utilized in the production of a wide range of products in these industries. 

Rose petals are rich in essential oil which contributes to the pleasant fragrance associated with roses [7]. Rose petal extracts or distillates (rose oils) are often incorporated into various cosmetic products, including perfumes, lotions, creams, and facial cleansers for their rich floral and appealing scent and potential skincare benefits [5,8]. Rose water, a by-product obtained during the steam distillation of rose petals, is commonly used in the cosmetic industry as a toner, facial mist, or ingredient in skincare products due to its hydrating and anti-inflammatory properties [9,10]. Rosehip seed oil, derived from rose hips is rich in sterols, tocopherols, and essential fatty acids and it is commonly used in skincare products for its nourishing and regenerative properties [11].

Rose petal extracts are also known for their potential health benefits, including antioxidant, anti-inflammatory, anti-elastase, analgesic, anticonvulsant, antimicrobial, and antidiabetic properties [12,13,14,15]. Thus, rose-derived compounds are used in the pharmaceutical industry in the formulation of diverse medicinal products and dietary supplements. Additionally, the fragrance of roses is often used in aromatherapy, contributing to stress relief and relaxation [16]. Roses are also edible plants that have been used in culinary applications for centuries. Recently, the concept of edible flowers has gained increased recognition, and roses are now utilized in the preparation of a variety of food products including jams, salads, ice creams, juices, and wines [17,18].

Considering all the information mentioned, the breeding and cultivation of roses with targeted chemical profiles, aiming to enhance their fragrance or provide significant amounts of health-beneficial compounds, is of utmost interest to the cosmetic and pharmaceutical industries. This is especially true for those sectors that prioritize the use of natural ingredients. 

In this study, petals of five novel garden rose genotypes, obtained through planned hybridization and distinguishing themselves by exhibiting exceptionally pleasant fragrances and large, intensely pink, violet, and red flowers, have been chosen for detailed examination of their chemical composition and biological activities to recommend them as potentially valuable candidates for use in cosmetic and pharmaceutical industries. Although the cultivar ‘Mileva Frayla’ possesses superior morphological characteristics, including the largest number of flowering laterals, the highest number of flowers per lateral, and relatively large petals, the selection must also take into account the biochemical profile, since it is responsible for the significant biological activities expressed by all cultivars explored in this study. Furthermore, the sensory analysis revealed different panelists’ and future consumers’ attitudes towards the fragrance components and their pleasantness. Fruity top notes, followed by flowery, fruity and rose-like middle components, supplemented with heavier base notes belonging to woody, musky, and balsamic, comprised unique petals’ aromas in all five investigated roses. Woody notes previously detected in peaches [19] tend to be positive attributes in consumers’ preferences, which was confirmed in our study with ‘Mina Frayla’. Complex compositions yielded interesting emotional associations with ‘Mina Frayla’ being scored as mild, light, fresh, intense, and warm. According to Wendin et al. [20] sensory analysis might be incorporated in consumer-oriented breeding and marketing of horticultural plants possessing unique fragrance compositions that might improve consumers’ health and well-being. Sensory analysis of edible *Begonia* × *tuberhybrida*, *Tropaeolum majus*, *Calendula officinalis*, *Rosa*, *Hemerocallis*, and *Tagetes patula* species, designed as ones with appropriately large flowers showed significant differences in appearance, fragrance, consistency, overall taste, and similar [21], marking the importance of all listed properties toward acceptance by consumers.

The petals of all investigated rose genotypes were very rich in phenolic compounds (TPC fell within the range from 148 mg GAE/g de to 260 mg GAE/g de). Plant phenolic compounds, also referred to as polyphenols, constitute a diverse group of naturally occurring chemicals characterized by their phenolic structure, featuring one or more phenol rings with hydroxyl groups. These compounds are extensively present throughout the plant kingdom and can be broadly categorized into four classes: phenolic acids, flavonoids, stilbenes, and lignans. Phenolic acids are further classified into hydroxybenzoic and hydroxycinnamic acids [22]. Polyphenols display a range of biological activities, encompassing antioxidant, anti-inflammatory, anti-cancer, and cardioprotective effects, thereby exhibiting potential health benefits [22]. The composition of polyphenols in plants can vary depending on factors such as plant type, variety, growing conditions, and maturity state [23]. The findings from previous examinations of methanol extracts from *R. brunonii*, *R. baurboniana*, and *R. damascena* (254 mg GAE/g de, 178 mg GAE/g de, and 145 mg GAE/g de, respectively) [24], methanol extracts from six edible cultivars of *R.* × *hybrida* (91.4–217 mg GAE/g de) [3], as well as ethanol extracts from nine cultivars of *R.* × *hybrida* (7.99–29.79 mg/g FW) [25], closely resembled the results obtained in this study. Notably, the ‘Andre Rieu’ cultivar exhibited even higher TPC than all other listed samples. It’s worth mentioning that the TPC in rose petals, particularly in the AR and PA samples, is comparable to that found in phenolic-rich fruits like blackberry and blueberry, while being much higher than levels observed in vegetables such as carrot and tomato [26].

Flavonoids, a diverse subgroup within the plant polyphenols, are characterized by the presence of two aromatic rings (A and B) connected by a chain of three carbon atoms. These compounds play a crucial role as pigments in flowers and fruits. Among them, anthocyanins stand out as a subgroup responsible for red, purple, blue, and violet tones. Beyond their function in providing vibrant colors, flavonoids, including anthocyanins, serve as effective absorbers of UV radiation and potent antioxidants, thereby shielding plants from the adverse effects of environmental stressors [27]. Numerous studies have underscored potent anti-inflammatory, anticancer, antimicrobial, and antiallergic properties of anthocyanins extracted from rose petals, making them valuable for applications in pharmaceuticals, functional foods, and cosmetics [1]. The TFC in the examined rose cultivars in this study was notably high, ranging from 23.7 mg QE/g de to 59.8 mg QE/g de. Comparable levels of TFC were previously identified in the ethanol extract of white rose (23.7 mg catechin equivalents per gram of dry extract) [14], methanol extracts of six edible cultivars of *R.* × *hybrida* (17.3–56.3 mg QE/g de) [3], and ethanol extracts of nine cultivars of *R.* × *hybrida* (0.786–5.31 mg catechin equivalents per gram of fresh weight) [25]. When examining the results of TFC and TPC, it is evident that the TFC for each sample is significantly less than its TPC. This indicates that only a minor fraction of the TPC is identified as flavonoids, while the extracts also contain substantial quantities of phenolic compounds from other categories.

Previous studies in the literature suggest that varieties with red and pink colors primarily consist of cyanidin glycosides, which are responsible for these specific hues [1]. In our study, where all investigated cultivars had purple, red, and violet-colored flowers, the TAC was high as expected, ranging from 2.21 to 13.1 mg CE/g. Samples AR and PA, exhibiting the most intense colors, were found to be the most abundant in anthocyanins. The TAC values of these samples significantly surpassed those values determined for pink/red/purple flowers of edible rose cultivars of *R.* × *hybrida* (‘Lavander Vaza’, ‘Marija Frayla’ and ‘Theo Clevers’) from our previous research (3.23–6.66 mg CE/g de) [3]. However, some rose varieties belonging to *R.* × *hybrida* exhibited anthocyanin levels as much as five times higher (5.03 mg CE/g fw), as demonstrated in a study by Yang et al. [25], compared to the richest cultivar in our study (AR), which recorded 1.31 mg CE/g fw.

LC-MS/MS quantitative analysis revealed that, among 45 investigated compounds, glycosides of quercetin (quercetin 3-*O*-glucoside, quercetin 3-*O*-galactoside, quercitrin and rutin), along with kaempferol-3-*O*-glucoside and quinic acid, stand out as dominant compounds in all examined rose cultivars. However, significant variations in the quantitative composition of these compounds were found among the samples, rather than differences in their qualitative aspects. The ‘Adore Aroma’ genotype exhibited the highest contents of the investigated flavonoid glycosides, while ‘Andre Rieu’ is characterized by the lowest content of these compounds along with the highest level of quinic acid. 

In a study by Mikanagi et al. [28], that investigated 120 taxa within subgenus *Rosa*,the prevalent flavonoids were recognized as kaempferol 3-*O*-glycosides and quercetin 3-*O*-glycosides. Listed findings align with the results of our study. However, the content of kaempferol 3-*O*-glucoside in six edible cultivars of *R.* × *hybrida* (60.9–193 μg/g de) in our previous research [3] was notably lower than in the cultivars investigated in this study (1147–23965 μg/g de).

Oxidative stress, triggered by an excessive generation of free radicals within the body, can exert harmful impacts on cells. This is due to the fact that these reactive free radicals have the potential to harm primary biomolecules such as lipids, proteins, carbohydrates, and nucleic acids, resulting in cellular dysfunction and the possibility of tissue and organ damage. Oxidative stress is implicated in a diverse array of health challenges, encompassing aging, as well as the initiation and progression of inflammatory conditions, atherosclerosis, and diverse chronic diseases. These conditions include cardiovascular diseases, neurodegenerative disorders such as Alzheimer’s and Parkinson’s, and cancer [29]. The consumption of antioxidants or their topical application stands as a preventive measure, capable of preventing or reducing oxidative stress and its associated adverse effects [30]. Antioxidants are commonly added to cosmetic formulations to help prevent unwanted chemical changes to the product that may occur due to oxidation. This protection against oxidation is crucial in maintaining the stability and quality of cosmetic formulations [31]. On the other hand, antioxidants in topical cosmetics help protect the skin against oxidative damage caused by free radicals and UV radiation [31]. This protective function is associated with slowing down the aging process. Nowadays, the use of plant-derived natural antioxidants in cosmetics is preferred over synthetic antioxidants [32]. Plant extracts generally contain a mixture of natural compounds, which could have synergetic effects; therefore, they can have better effects and less toxicity than synthetic antioxidants [32]. 

Both assays (DPPH and FRAP) employed in the present study to assess the antioxidant potential of rose petal extracts are based on a single electron transfer mechanism. In the DPPH assay, antioxidants convert the DPPH radical into a stable molecule, while in the FRAP assay, they reduce ferric ion (Fe^3+^) to ferrous ion (Fe^2+^). Simultaneously, the antioxidants are transformed into a relatively stable and unreactive radical form. The rose petal extracts from all examined genotypes exhibited significant antioxidant activity, surpassing the efficacy of vitamin C, a recognized potent antioxidant with an IC_50_ of 30.0 µg/mL in the DPPH assay [24], and slightly lower than the synthetic antioxidant BHA (IC_50_ of 11.08 µg/mL), extensively used in the food and cosmetic industries [33]. The extracts’ activity was comparable to that of previously tested methanol extracts from *R. brunonii*, *R. baurboniana*, and *R. damascene* (with IC_50_ values in the DPPH assay being 35.2 µg/mL, 25.0 µg/mL, and 21.4 µg/mL, respectively) [24]. Since new rose genotypes investigated in this study exhibited high antioxidant activity, they are promising candidates for inclusion in cosmetic products and pharmaceutical formulations. AChE catalyzes the hydrolysis of acetylcholine (ACh), an endogenous neurotransmitter, converting it into acetic acid and choline. Thus, AChE serves to terminate synaptic transmission in chemical synapses of the cholinergic type within the central nervous system, autonomic ganglia, and neuromuscular junctions. Reduced concentrations of AChE are frequently noted in neurodegenerative disorders like Alzheimer’s disease and various forms of dementia. Inhibition of AChE leads to enhancement of Ach level within synapse, consequently increasing cholinergic signaling and enhancing cognitive functions, encompassing learning, memory, behavior, and emotional responses. Therefore, reversible inhibitors of AChE, such as galantamine, rivastigmine, and donepezil are often used in treatments of dementia. However, these drugs could have detrimental adverse effects including gastrointestinal issues, fatigue, cramps, and sinus node dysfunction [34]. Hence, scientists are persistently searching for novel AChE inhibitors that not only effectively inhibit the enzyme, but also come with fewer unwanted side effects. Various plant phenolics were proven to successfully inhibit AChE, thus having neuroprotective activity [35]. The findings of this investigation validate these results, as the substantial correlation coefficient (R^2^ = 0.9948) observed between TPC and anti-AChE activity in rose extracts suggested that a higher polyphenolic content significantly contributes to enhanced anti-AChE activity. Since these compounds, beside AChE inhibitory activity, often exhibit additional pharmacological properties, especially antioxidants, they could be applied in multi-target strategies for combating the onset and progression of Alzheimer’s disease and other neurological conditions. The AChE inhibitory activity of new rose genotypes investigated in this study was moderate (29–40%) at a concentration of 50 µg/mL, with the ‘Pure Aroma’ genotype exhibiting the highest activity. The PA extract stands out from others due to significantly higher content of quercitrin, indicating that this compound could contribute to the activity. However, the activity of PA is still lower than the activity of ‘Eveline Wide’ genotype of *R* × *hybrida* (69.4%) investigated in our previous study [3]. In comparison to the activity of different varieties of *R. damascena* from Turkey (IC_50_ values in the range from 3.9 μg/mL to 32.0 μg/mL), new cultivars investigated in the present study showed comparable activity.

Diabetes mellitus (DM) is a chronic metabolic disease characterized by elevated blood glucose levels, which has become a widespread health concern worldwide. It is considered as a top 10 cause of death globally, contributing to approximately 1.6 million deaths [36]. Prolonged hyperglycemia results in increased generation of reactive oxygen species (ROS) and consequently, the onset of oxidative stress and inflammation. These conditions are implicated in the impairment of the pancreatic β-cells and DM complications [37]. One of the strategies for lowering blood glucose levels, which is used in the treatment of DM, is slowing down the absorption of carbohydrates following food intake. This can be achieved by inhibiting the activity of α-amylase and α-glucosidase, the enzymes in the gastrointestinal tract involved in the breakdown of complex carbohydrates into simpler sugars (such as glucose) during the digestive process [37]. Inhibitors of α-glucosidase and α-amylase, such as acarbose, miglitol, and voglibose, are often used in the management of DM. However, these drugs have unwanted side effects, such as flatulence, diarrhea, and abdominal distention and pain, due to bacterial action on undigested carbohydrates [38]. Extracts of new rose genotypes investigated in this study expressed extremely high inhibitory potential to α-glucosidase, significantly higher than that of acarbose, and negligible activity against α-amylase.Even at a very low concentration (2.5 µg/mL), samples PA and AR achieved nearly 90% inhibition of α-glucosidase, while samples AA, MIL, and MIF exhibited slightly lower inhibitory activity (47.2–57.1%). Moreover, the results of this study suggest that among the investigated polyphenol types, anthocyanins exhibit the highest potential for inhibiting α-glucosidase, as opposed to phenols and flavonoids. Specifically, the correlation coefficient observed between TAC and anti-α-glucosidase activity (R^2^ = 0.8748) was considerably higher than those observed for TPC and TFC and anti-α-glucosidase activity (R^2^= 0.7634 and R^2^ = 0.3568, respectively). In the study of Gholamhoseinian et al. [39], methanol extract of the flowers of *R. damascene* expressed anti-α-glucosidase activity in vitro (at a concentration of 2 µg/mL inhibited AchE for 98%), comparable to the activity of AR and PA genotypes in the present study. In the same study [39], the activity of *R. damascene* extract was confirmed in vivo (in normal and diabetic rats), where the extract significantly suppressed the elevation of blood glucose after the administration of the high maltose diet. This indicates the great potential of *R. damascene*, as well as the extracts of new genotypes investigated in the present study, to be used to suppress postprandial hyperglycemia in diabetic patients, making them good candidates for the development of new formulations for alternative and/or complementary management of Diabetes mellitus type 2. They could also be applied to control obesity by reducing the food efficiency ratio, particularly the intake of carbohydrates.

## 4. Materials and Methods

### 4.1. Plant Material 

The plant material utilized in the experiments (depicted in Figure 1) comprised five tetraploid (4n) garden rose cultivars named ‘Pure Aroma’ (PA), ‘Adore Aroma’ (AA), ‘Andre Rieu’ (AR), ‘Mina Frayla’ (MIF), and ‘Mileva Frayla’ (MIL), predominantly available on market in Serbia, Hungary, Poland, France, Italy, Germany, Netherlands, and the UK. All these genotypes belong to the *R* × *hybrida* species and were cultivated using conventional breeding practices, devoid of any chemical protection.

The garden rose specimens were of a biennial age and thrived in the outdoor environmental settings at the ‘Pheno Geno Roses’ private company in Temerin, Northern Serbia (coordinates: 45°24′19″ N 19°53′13″ E/45.105166° N 19.886833° E). The area is distinguished by a conventional continental climate, featuring notably warm summers and frigid winters.

The research field, measuring 30 m in length and 20 m in width, was created during the autumn of 2017 through on-site bud grafting. There were 150 grafted plants for each cultivar, with a 10 cm spacing between plants and a 1 m gap between rows.

In late August 2021, forty flowers from each cultivar were gathered. The freshly picked blooms were preserved by freezing at −80 °C until subjected to analysis. 

### 4.2. Morphological Traits 

The morphological characterization during the full blossom included both descriptive and metrical properties following the UPOV protocol [40] for roses (*Rosa* L.). The methodology was previously described in detail by Simin et al. [3].

### 4.3. Sensory Analysis

Fragrance evaluation as a qualitative trait was performed by 45 panel specialists (of different genders, seniority, specialties, and interests in roses). Detailed methodology regarding fragrance components divided into top, heart, and base notes, smelling time, and replications, as well as data approximation was published by Simin et al. [3]. 

### 4.4. Preparation of Methanol Extracts

Methanol extracts of rose petals were prepared via maceration of frozen plant material with 80% MeOH (in a 1:10 ratio) over 48 h at room temperature. The resulting macerate underwent filtration, and the maceration process was repeated once more. Subsequently, the macerates were evaporated to dryness under vacuum at 35 °C. The resulting dry extracts were dissolved in DMSO to reach a final concentration of 200 mg/mL and were stored at −20 °C until the time of analysis. The obtained extracts were used for chemical composition analysis and assessing biological activities, including antioxidant, neuroprotective, and antidiabetic.

### 4.5. Chemical Characterization of Rose Petal Methanol Extracts

#### 4.5.1. Determination of Total Phenolic Content

TPC was determined by Folin–Ciocalteu (FC) assay, described previously by Lesjak et al. [41].

#### 4.5.2. Determination of Total Flavonoid Content

Determination of TFC was conducted using the aluminum chloride assay method as outlined by Lesjak et al. [41]. 

#### 4.5.3. Determination of Total Monomeric Anthocyanin Content

The determination of TAC was carried out utilizing the pH differential method, following the procedure previously published [42], with adaptations suitable for 96-well microplates.

#### 4.5.4. Quantitative Analysis of Selected Compounds

The content of quinic acid and 44 selected phenolic compounds (14 phenolic acids, 25 flavonoids, three coumarins, and two lignans) was investigated using LC-MS/MS according to the previously described method [33]. 

### 4.6. Antioxidant Potential

#### DPPH and FRAP Assays

The capability of the extracts to counteract the DPPH radical was assessed following the method outlined by Lesjak et al. [41]. Furthermore, FRAP assay was conducted following the procedure also described by Lesjak et al. [41].

### 4.7. Neuroprotective Activity 

The neuroprotective potential of the extracts was evaluated by determining AChE-IP through Ellman’s method, with specific modifications detailed in a previous study by Pintać et al. [43].

### 4.8. Antidiabetic Activity

The antidiabetic activity was examined by measuring the potential to inhibit α-amylase and α-glucosidase. 

Inhibition of α-amylase was examined with a method used by Yang et al. [44], adapted for a 96-well plate. Briefly, 90µL of alpha-amylase (0.1 µg/mL in 20 mM phosphate buffer pH 6.9) were added to 80µL 0.05% starch dissolved in 20 mM phosphate buffer pH 6.9 along with 10 µL of extract (5.0 mg/mL) or standard (acarbose, 0.125–3.0 mg/mL). In the blank probe, α-amylase was replaced with 20 mM phosphate buffer pH 6.9, and in the control 20 mM phosphate buffer pH 6.9 was added instead of the sample. The 96-well plate was then incubated for 10min at 37 °C with constant shaking. After incubation, the reaction was stopped by the addition of 100 µL of cold 1 M HCl, after which, 20 µL of Lugol solution was added and absorbance was measured at 620 nm. All tests were done in triplicate and results were expressed as % of inhibition and mg of ACAE/g de.

Inhibition of α-glucosidase was examined using a modified procedure of Palanisamy et al. [45]. Briefly, 100 µL of 0.1 M phosphate buffer pH 6.8 was mixed with 10 µL of α-glucosidase (0.1 U/mL in 0.1 M phosphate buffer pH 6.8), 20 µL of extract (20 µg/mL) or standard (acarbose, 0.310–10.0 mg/mL), and 20 µL of *p*-nitrophenyl α-D-glucoside. In the blank probe, α-glucosidase was substituted with 0.1 M phosphate buffer pH 6.8, and in the control, instead of the sample, 20 µL of 0.1 M phosphate buffer was added. The 96-well plate was then incubated for 15min at 37 °C with constant shaking. After incubation for 15 min, 80 µL of 0.2 M Na_2_CO_3_ and absorbance was measured at 400 nm. All tests were done in triplicate and the results were expressed as % of inhibition and g ACAE/g de.

### 4.9. Statistical Analysis 

The data of all spectrophotometric measurements were analyzed using one-way ANOVA followed by the post hoc Tukey’s Honest Significant Difference (HSD) test for multiple comparisons of means in order to determine whether the data obtained for different rose genotypes differed significantly between each other (Real Statistics Resource Pack add in for Excel 2013). Statistical significance was set at *p*< 0.05. Principal component analysis (PCA) and hierarchical clustering analysis of results of LC-MS/MS analysis was done using Past version 4.11.

## 5. Conclusions

This intersection of horticulture and industry showcases the potential for innovation in creating roses that not only serve ornamental purposes but also contribute to the formulation of diverse and beneficial products in the cosmetic and pharmaceutical fields. The objective of this research was to further enhance the value of these rose genotypes by analyzing the composition of biologically active polyphenols and exploring potential health benefits. Morphologically, the cultivar MIL is distinguished by having the largest number of flowering laterals, the highest number of flowers per lateral, and relatively large petals, but the significant biological activities were expressed by all cultivars explored in this study.

The petals of all investigated rose genotypes were very rich in phenolic compounds (TPC ranging between 148 mg GAE/g de and 260 mg GAE/g de). Furthermore, LC-MS/MS quantitative analysis revealed that, among 45 investigated compounds, glycosides of quercetin (quercetin 3-*O*-glucoside, quercetin 3-*O*-galactoside, quercitrin and rutin), along with kaempferol-3-*O*-glucoside and quinic acid, were dominant compounds in all examined rose cultivars. Additionally, the extracts of all investigated genotypes expressed very high antioxidant activity, much higher than the activity of vitamin C, which is known as a potent antioxidant (IC_50_ in DPPH assay is 30.0 µg/mL). Due to the very high antioxidant activity observed for methanol extracts of petals from the new rose genotypes investigated in this study, they could be recommended as promising candidates for application as an ingredient of cosmetic products, as well as of pharmaceutical formulations. 

One particularly important result was the AChE inhibitory activity (29–40%) at a concentration of 50 µg/mL, with the ‘Pure Aroma’ genotype exhibiting the highest activity. The PA extract stands out from others due to the significantly higher content of quercitrin, indicating that this compound could contribute to neuroprotective activity. Extracts of new rose genotypes investigated in this study expressed extremely high inhibitory potential to α-glucosidase, significantly higher than that of acarbose, and negligible activity against α-amylase. Even at a very low concentration (2.5 µg/mL), samples PA and AR achieved nearly 90% inhibition of α-glucosidase, while other samples exhibited slightly lower inhibitory activity (47.2–57.1%). This indicates great potential of the investigated genotypes to be used as anti-diabetic agents.

Due to their valuable health-promoting properties, the studied new rose genotypes could potentially act as drugs in the prophylaxis and treatment of many diseases. A great challenge for future research in pharmacology and medical fields could be the study of these roses in preclinical and clinical trials.

## Figures and Tables

**Table 1:** Investigated vegetative characteristics of five rose genotypes.

Cultivar/Trait	PA	AA	AR	MIF	MIL
**Plant**					
GT	Shrub	Shrub	Shrub	Shrub	Shrub
GH	Semi-upright	Upright	Upright	Upright	Semi-upright
Height (cm)	80.8 ^b^	69.6 ^d^	75.0 ^c^	79.8 ^b^	90.4 ^a^
**Leaf**					
IGC	Dark	Dark	Medium	Dark	Medium
LAC	Present	Present	Present	Present	Present
GUS	Weak	Medium	Weak	Medium	Very weak
Length (cm)	4.76 ^b^	4.24 ^c^	4.56 ^b^	3.58 ^d^	5.68 ^a^
Width (cm)	2.98 ^b^	2.86 ^b,c^	3.72 ^a^	2.66 ^c^	3.88 ^a^

Glossiness of upper side—GUS; growth habit (excluding climbers)—GH; growth type—GT; intensity of green color (upper side)—IGC; leaf anthocyanin coloration—LAC; PA—‘Pure Aroma’; MIF—‘Mina Frayla’; AA—‘Adore Aroma’; AR—‘Andre Rieu’; MIL—‘Mileva Frayla’. Mean values designated with the same letter were not significantly different according to Tukey’s HSD test (*p* ≤ 0.05).

**Table 2:** Flowering characteristics of the analyzed rose genotypes.

Cultivar/Trait	PA	AA	AR	MIF	MIL
**Flowering Shoot**					
NFS	0 ^b^	1.82 ^a^	0 ^b^	0 ^b^	0 ^b^
FL	Present	Absent	Present	Present	Present
NFL	1.83 ^b^	0 ^d^	1.64 ^c^	1.62 ^c^	3.44 ^a^
NF/L	2.94 ^c^	0 ^d^	3.86 ^b^	3.68 ^b^	8.72 ^a^
**Flower**					
TP	Double	Double	Double	Double	Double
CG	Medium purple	Medium violet	Dark purple	Medium purple red	Medium purple red
CC	Red purple	Red purple	Red purple	Red purple	Pink
SH	Irregularly rounded	Rounded	Rounded	Irregularly rounded	Rounded
PUP	Flat	Flat	Flat	Flat	Flat
PLP	Concave	Concave	Concave	Concave	Flat
FG	5	5	5	5	5
NOP	96.2 ^c^	114.4 ^a^	107.4 ^b^	65.8 ^d^	94.4 ^c^
DM (cm)	5.86 ^b^	6.54 ^a^	6.66 ^a^	6.50 ^a^	6.56 ^a^
PL (cm)	4.40 ^b^	4.48 ^b^	4.84 ^a^	3.72 ^c^	3.32 ^d^
PW (cm)	4.24 ^a^	3.58 ^b^	3.36 ^b^	3.39 ^b^	3.24 ^b^

Color group—CG; Color of center (only varieties with flower type double)—CC; Flower diameter—DM; Flowering laterals—FL; Fragrance—FG on the 1–5 scale; Number of flowering laterals—NFL; Number of flowers per lateral (Only varieties with flowering laterals)—NF/L; Number of flowering shoots (Only varieties with no flowering laterals)—NFS; Number of petals—NOP; Profile of lower part—PLP; Shape—SH; Petals length—PL; Petals width—PW; Profile of upper part—PUP; type—TP; Mean values designated with the same letter were not significantly different according to Tukey’s Honest Significant Difference test (*p* ≤ 0.05).

**Table 3:** Contents of total phenolics, total flavonoids, and total anthocyanins in rose petal methanol extracts.

	TPC		TFC		TAC	
Genotype	mg GAE/g de	mg GAE/g fw	mg QE/g de	mg QE/g fw	mg CE/g de	µg CE/g fw
PA	249 ± 13.0 a ^a^	26.8 ± 1.40 a	43.7 ± 2.52 b	4.70 ± 0.271 c	9.19 ± 0.193 b	0.990 ± 0.021 b
AA	148 ± 14.6 c	14.0 ± 1.31 c	59.8 ± 3.73 a	5.65 ± 0.335 b	7.55 ± 0.317 c	0.714 ± 0.028 c
AR	260 ± 5.01 a	25.9 ± 0.50 a	23.7 ± 0.215 c	2.36 ± 0.021 e	13.1 ± 0.615 a	1.31 ± 0.061 a
MIF	182 ± 15.8 bc	18.2 ± 1.58 b	56.8 ± 4.90 a	5.70 ± 0.492 a	2.24 ± 0.019 d	0.225 ± 0.002 d
MIL	193 ± 18.3 b	16.4 ± 1.56 bc	44.0 ± 2.81 b	3.75 ± 0.239 d	2.21 ± 0.008 d	0.188 ± 0.001 e

^a^ values are means ± standard error. Means within each column with different letters (a–e) differ significantly according to Tukey’s HSD test (*p* ≤ 0.05);AA—‘Adore Aroma’; AR—‘Andre Rieu’; CE—cyanidin-3-*O*-glucoside equivalents; de—dry extract; fw—fresh weight; GAE—gallic acid equivalents; MIF—‘Mina Frayla’; MIL—‘Mileva Frayla’; PA—‘Pure Aroma’; QE—quercetin equivalents; TAC—total anthocyanin content; TFC total flavonoid content; TPC total phenolic content.

**Table 4:** Contents of quinic acid and selected phenolic compounds in methanol extracts of rose petals.

	Content [μg/g de] ^a^				
Genotype	PA	AA	AR	MIF	MIL
Quinic acid	**16,837 ^b^** ± 1684 bc ^c^	**13,380** ± 1338 c	**23,929** ± 2393 a	**14,960** ± 1496 c	**21,541** ± 2154 ab
Protocatechuic acid	**43.9** ± 3.51 b	**60.7** ± 4.86 a	**51.6** ± 4.13 b	**15.3** ± 1.22 c	**12.8** ± 1.02 c
*p*-Coumaric acid	0.209 ± 0.019 b	2.92 ± 0.262 a	0.111 ± 0.010 b	0.137 ± 0.012 b	0.173 ± 0.016 b
Gallic acid	**22.6** ± 2.03 cd	**29.3** ± 2.64 bc	**21.7** ± 1.95 d	**30.4** ± 2.74 ab	**36.4** ± 3.27 a
Naringenin	0.335 ± 0.023 b	0.717 ± 0.050 a	0.651 ± 0.046 a	<0.3 ^d^	0.341 ± 0.024 b
Luteolin	0.520 ± 0.026 a	0.523 ± 0.026 a	0.546 ± 0.027 a	0.520 ± 0.026 a	0.440 ± 0.022 b
Kaempferol	2.48 ± 0.174 c	9.52 ± 0.666 a	1.61 ± 0.113 c	5.79 ± 0.405 b	6.41 ± 0.448 b
Catechin	**19.2** ± 1.92 b	**144** ± 14.4 a	**133** ± 13.3 a	**30.9** ± 3.09 b	**26.5** ± 2.65 b
Epicatechin	0.969 ± 0.097 c	1.92 ± 0.192 b	5.17 ± 0.517 a	/ ^e^	/
Chrysoeriol	0.171 ± 0.005 c	< 0.075	0.170 ± 0.005 c	0.960 ± 0.029 b	2.10 ± 0.063 a
Quercetin	**31.4** ± 9.43 ab	**59.2** ± 17.8 a	**53.3** ± 16.0 a	**14.9** ± 4.48 b	**17.1** ± 5.14 b
Chlorogenic acid	0.603 ± 0.030 d	1.37 ± 0.069 c	0.502 ± 0.025 d	3.02 ± 0.151 b	5.98 ± 0.299 a
Apigenin- 7-*O*-glucoside	<0.075	0.131 ± 0.007a	0.116 ± 0.006 b	<0.075	<0.075
Vitexin	<0.075	<0.075	<0.075	0.102 ± 0.005 b	0.132 ± 0.007 a
Kaempferol- 3-*O*-glucoside	**2348** ± 93.9 d	**23,965** ± 959 a	**1147** ± 45.9 d	**11,454** ± 458 c	**14,275** ± 571 b
Luteolin- 7-*O*-glucoside	1.10 ± 0.033 a	/	0.824 ± 0.025 b	/	/
Quercitrin	**13,945** ± 837 a	**10,654** ± 639 b	**2978** ± 179 d	**9338** ± 560 bc	**8564** ± 514 c
Quercetin- 3-*O*-glucoside + Quercetin- 3-*O*-galactoside	**8267** ± 496 d	**19,949** ± 1197 a	**3981** ± 239 e	**11,969** ± 718 c	**14,709** ± 883 b
Rutin	**2235** ± 67.0 b	**2193** ± 65.8 b	**703** ± 21.1 c	**2188** ± 65.6 b	**2663** ± 79.9 a
Total phenolics (mg/g de) ^f^	43.76	70.45	33.01	50.01	61.86

^a^ Results are given as content (μg/g of dry extract) ± standard error of repeatability (as determined via method validation); ^b^ The values higher than 10 are marked with bold letters; ^c^ means within each row with different letters (a–e) differ significantly according to Tukey’s HSD test (*p* ≤ 0.05); ^d^ below limit of quantitation (LoQ); ^e^ not detected; ^f^ Sum of the contents of all detected phenolic compounds using LC-MS/MS; AA—‘Adore Aroma’; AR—‘Andre Rieu’; de—dry extract; MIF—‘Mina Frayla’; MIL—‘Mileva Frayla’; PA—‘Pure Aroma’.

**Table 5:** Antioxidant, neuroprotective, and antidiabetic activity of rose petal methanol extracts.

	FRAP	DPPH IC_50_	AChE-IP	α-Amylase-IP		α-Glucosidase-IP	
Genotype	mg AAE /g de	μg/mL	ng EE/g de	I (%)	mg ACAE /g de	I (%)	g ACAE /g de
**PA**	209 ± 10.1a ^a^	18.6 ± 1.25 c	18.3 ± 0.561 a	11.5 ± 1.62 a	95.6 ± 13.6 a	87.1 ± 4.11 a	292 ± 7.20 b
**AA**	132 ± 7.40 d	27.8 ± 1.01 a	10.2 ± 0.117 e	2.40 ± 0.435 b	19.5 ± 3.66 b	47.2 ± 4.35 b	38.6 ± 6.98 d
**AR**	198 ± 12.8 ab	22.7 ± 0.16 b	16.4 ± 0.405 b	13.6 ± 1.99 a	113 ± 16.7 a	86.3 ± 1.19 a	339 ± 39.4 a
**MIF**	175 ± 6.24 c	24.0 ± 1.19 b	12.4 ± 0.132 d	0.94 ± 0.156 c	7.3 ± 1.32 c	57.1 ± 5.70 b	60.7 ± 16.4 c
**MIL**	177 ± 1.96 bc	17.7 ± 1.27 c	14.7 ± 1.16 c	0.00 ± 0.00 d	0.00 ± 0.00 d	54.5 ± 1.01 b	52.7 ± 2.39 c

^a^ means within each row with different letters (a–e) differ significantly according to Tukey’s HSD test (*p* ≤ 0.05); AA—‘Adore Aroma’; AAE—ascorbic acid equivalents; ACAE—acarbose equivalents; AChE-IP—the potential to inhibit acetylcholinesterase; AR—‘Andre Rieu’; de—dry extract; EE—eserine equivalents; FRAP—Ferric reducing antioxidant potential; I—inhibition; IC_50_ (DPPH)—the concentration of the extract that neutralizes 50% of DPPH radicals; MIF—‘Mina Frayla’; MIL—‘Mileva Frayla’; PA—‘Pure Aroma’; α-amylase-IP—the potential to inhibit α-amylase; α-glucosidase-IP—the potential to inhibit α-glucosidase.

## Data Availability

The datasets used and/or analyzed during the current study are available from the corresponding author on a reasonable request.
